# Supplementary material for: Simplified Generation of Biomedical 3D Surface Model Data for Embedding into 3D Portable Document Format (PDF) Files for Publication and Education
Source: PLoS One. 2013 Nov 15;8(11):e79004. doi: 10.1371/journal.pone.0079004 (PMC3829830; doi:10.1371/journal.pone.0079004)
Supplement: File S2 — Supplementary version of this article with embedded 3-d figures. (PDF) [file pone.0079004.s002.pdf]

Simplified Generation of Biomedical 3D Surface Model Data for Embedding into 3D Portable Document Format (PDF) Files for Publication and Education

Axel Newe[1]*, Thomas Ganslandt[2]

1 Chair of Medical Informatics, Friedrich-Alexander-University Erlangen-Nuremberg, Erlangen, Germany, 2 Medical Centre for Information and Communication Technology, University Hospital Erlangen, Erlangen, Germany

Abstract

The usefulness of the 3D Portable Document Format (PDF) for clinical, educational, and research purposes has recently been shown. However, the lack of a simple tool for converting biomedical data into the model data in the necessary Universal 3D (U3D) file format is a drawback for the broad acceptance of this new technology. A new module for the image processing and rapid prototyping framework MeVisLab does not only provide a platform-independent possibility to create surface meshes out of biomedical/DICOM and other data and to export them into U3D – it also lets the user add meta data to these meshes to predefine colors and names that can be processed by a PDF authoring software while generating 3D PDF files. Furthermore, the source code of the respective module is available and well documented so that it can easily be modified for own purposes.

Citation: Newe A, Ganslandt T (2013) Simplified Generation of Biomedical 3D Surface Model Data for Embedding into 3D Portable Document Format (PDF) Files for Publication and Education. PLoS ONE 8(11): e79004. doi:10.1371/journal.pone.0079004

Editor: Peter M.A. van Ooijen, University of Groningen, University Medical Center Groningen, Netherlands

Received July 23, 2013; **Accepted** September 25, 2013; **Published** November 15, 2013

Copyright: © 2013 Newe, Ganslandt. This is an open-access article distributed under the terms of the Creative Commons Attribution License, which permits unrestricted use, distribution, and reproduction in any medium, provided the original author and source are credited.

Funding: The authors acknowledge support by Deutsche Forschungsgemeinschaft (DFG) and Friedrich-Alexander-University Erlangen-Nuremberg within the funding programme Open Access Publishing. The funders had no role in study design, data collection and analysis, decision to publish, or preparation of the manuscript.

Competing Interests: The authors have declared that no competing interests exist.

* E-mail: axel.newe@fau.de

Introduction

The Portable Document Format with Embedded 3D Models

The Portable Document Format (PDF) is a comprehensive document description language used to define electronic documents independently of its creating, displaying and printing software, hardware and operating system. A PDF file encapsulates all resources to completely describe the content and layout of an electronic document, including texts, fonts, images and multimedia elements without the need of external resources.

Starting with version 1.6 of the PDF Specification [1], implemented and published first in 2007 with Adobe Acrobat 3D Version 8 and Adobe Reader 8.1, three-dimensional mesh models can be embedded into this widely known and used file format (more than 500 million users worldwide, according to Adobe: http://www.adobe.com/uk/pdf/), which has been the de-facto standard for the exchange of electronic documents for years now. An alternative is not in sight.

The Adobe Reader (http://get.adobe.com/reader/otherversions/) offers many options to display these mesh models (solid surface, transparent surface, wireframe, point cloud, contour lines, illumination) and to let the user interact with them (zooming, panning, rotating, selection of components). Using embedded scripting, even complex animations and interaction with other components (e.g. form elements) of the respective PDF document are possible.

Several authors have proven these 3D models embedded into PDF documents to be useful for electronic publication in biology [2,3], (bio-)chemistry [4,5,6,7] and medicine [8,9,10,11,12] and superior over alternative solutions. Spatial relationships (like the vessel systems in the liver or neuronal fibers in the central nervous system) can easily be differentiated and perceived much better than by textual description [2,13]. The consumer of a document is not dependent on the one single view the author has selected for a 3D scene, but can freely decide which view(s) shall be used for a printout, based on his own preferences or interests. Furthermore, the interaction aspect might be a trigger for a detailed exploration driven by the reader's curiosity [2]. Even journals start requesting their authors to embed multimedia content directly into their publications [14], because the former concept of supplemental external resources undermines the concept of a completely self-contained document with all necessary information [15].

Besides that, it is a simple fact, that much of raw data in science is 3D by its nature: molecules, microscopic and macroscopic anatomy, propagation of radiation – traditional ways of presenting this kind of data in 2D come with an inherent loss of information. No 2D image, illustration, stereograph or descriptive text will ever describe 3D data as precisely and in full extent as a 3D representation can do and therefore should.

Simplifying the Generation of U3D Model Data

The generation of the necessary mesh model data is still cumbersome. Previous authors needed a tool chain of at least three [6,8] or even four [11] different software applications and up to 22

single steps until the final PDF was created. Furthermore, some of these tools are not available for all platforms (OsiriX only for MacOS, used by [11]), are commercial software with closed source and license costs (Amira and Adobe 3D Toolkit, used by [2]) or need intermediate file formats and processing steps (MeshLab, used by [6] & [8]).

The replacement of the last tool in this chain is not reasonable. Some kind of PDF authoring tool will always be needed since it cannot be expected that an application that generates 3D scene data also provides the ability to set text layout, process screenshots etc. Therefore a one-click-solution as discussed by [6] is not really feasible, but the number of tools should be reduced to a maximum of two applications: one for generating the 3D scene data and one for generating the final PDF. In this paper, we present a novel way to create this scene data.

Background and Related Work

PDF Features and Suitability for Biomedical Documents

The PDF specification (latest version 1.7, extension level 5) is very well documented and available to the full extent from its developer Adobe (http://www.adobe.com/devnet/pdf/pdf_reference.edu.html). The usage is free of charge, as well as the Adobe Reader that is available for all major operating systems (MS Windows, Mac OS, Linux) and currently the only software for displaying and printing PDF documents that fully supports all features of PDF (including multimedia and 3D). Adobe Acrobat is the Reader's commercial counterpart for creating and editing PDF documents. Although there are many commercial and free tools available for creating PDF files or for converting other documents into PDF, Adobe Acrobat is the only off-the-shelf software that fully supports all PDF features (especially regarding 3D models: http://convert-pdf-software-review.toptenreviews.com/). Besides that, it is also available for Windows, Mac OS and Linux.

PDF specification 1.7 is also published by the International Organization for Standardization as ISO 32000–1:2008 [16] and fulfills all requirements for an interactive publication document as postulated by Thoma et. al. [15].

A general major issue regarding the exchange of medical data is privacy and security. PDF provides the possibility to encrypt documents (with AES or RC4) and to sign them digitally. Although [17] has proved that PDF security is not waterproof in all respects, the contents of PDF documents themselves could not be disclosed. This makes PDF documents suitable for the exchange of medical data. In 2008, the Association for Information and Image Management (AIIM) has released a Best Practice Guide for the implementation of PDF in healthcare (AIIM BP02–2008), also known as PDF/H (http://www.aiim.org/Research-and-Publications/Standards/Articles/PDF-Healthcare, [18]), that is officially accepted by Adobe [19].

In addition to that, DICOM Supplement 104: "DICOM Encapsulation of PDF Documents" [20] defines a SOP Class to encapsulate PDF documents into a Composite DICOM SOP Instance using the Secondary Capture object, so that PDF files can be exchanged using the appropriate DICOM Service Classes.

Caveats regarding the PDF format with embedded 3D models discussed by other authors (e.g. [2]) are almost obsolete. Long-time compatibility and readability should be solved with the transfer of the PDF specification to ISO 32000. Even simple desktop hardware is nowadays capable of displaying interactive 3D scenes. In the case that processing power is not sufficient for a smooth rendering, Adobe Reader dynamically reduces details during the interaction and renders again with full details right after the interactive manipulation of the respective scene has ended. The only hardware that is currently not capable of rendering 3D scenes is the growing field of tablet computers.

The Universal 3D (U3D) File Format

PDF allows importing two different 3D model file formats: the Product Representation Compact (PRC) format and the Universal 3D (U3D) format. Although PRC is the older format (first appearance around 2002) and published as ISO 14739-1, U3D seems to have become more accepted and is nowadays available as export format for many software applications dealing with 3D models. It was initially defined as an exchange format for 3D model data in Computer Aided Construction (CAD) by a consortium of companies related to this industry (including e.g. Intel, Siemens and Boeing). In December 2004, the Ecma International (formerly known as European Computer Manufacturers Association, ECMA) published the first edition of its standard ECMA-363 (Universal 3D File Format); the latest version is the 4th edition from June 2007 [21].

Universal 3D is a binary file format that contains all necessary information to describe a 3D scene graph. This includes the geometry data, palette definitions, lighting, cameras ("views"), texturing and pre-defined animations ("motions").

A U3D scene consists of an arbitrary number of objects that can be sorted in a monohierarchic object tree. The geometry of each object can be defined as a triangulated surface mesh, a set of lines or a set of points ("point cloud"). For smooth rendering, the level of detail can be defined depending on the distance to the viewpoint (CLOD – Continuous Level of Detail). A proprietary bit encoding algorithm allows for a highly compressed storage of the geometry data. The possibility to re-use resources once defined (e.g. objects of the same geometry with different colors) further contributes to the reduction of the final file size [21].

U3D files are sequences of "blocks", always starting with a "File Header Block" (block type 0x00443355, which reads as "U3D" in ASCII). The File Header Block is followed by "Declaration Blocks" and "Continuation Blocks". Declaration Blocks contain information about the objects (e.g. mesh definitions or texture resources) in the file and Continuation Blocks can provide additional information for objects declared in a Declaration Block (e.g. the vertex coordinates of a mesh) [21].

Materials and Methods

A New Module for MeVisLab

To achieve the goal of simplifying the creation of U3D files by reducing the number of necessary tools to only one application in (but not limited to) the field of biomedical image processing, a new module for MeVisLab (http://www.mevislab.de/) was created. MeVisLab is an image processing framework and visual development environment, developed by MeVis Medical Solutions AG and Fraunhofer MEVIS (formerly MeVis Research GmbH) in Bremen, Germany. It is available for all major platforms (MS Windows, Mac OS and Linux: http://www.mevislab.de/download/) and offers a variety of licensing options, including a "MeVisLab SDK Unregistered" license which is free for use in non-commercial organizations and research (http://www.mevislab.de/mevislab/versions-and-licensing/). MeVisLab can not only be used as a toolbox for simple image processing, but also as a framework for creating sophisticated applications with graphical user interfaces that hide the underlying platform and do not require substantial programming knowledge [22,23,24]. The general usage of MeVisLab is explained in its comprehensive and easy-to-understand documentation (http://www.mevislab.de/developer/documentation/). Especially the "Getting Started

Tutorial'' is recommended to be perused by newcomers. It is available for direct download (http://www.mevislab.de/fileadmin/docs/current/MeVisLab/Resources/Documentation/Publish/SDK/GettingStarted.pdf) as well as with the MeVisLab installation (Menu ''Help'' → ''Show Help Overview'' → ''Getting Started'').

The modular design of MeVisLab allows for simple assembling of image processing ''networks'' [24] and comes with more than 800 pre-defined standard components (''modules''). About 1800 additional modules completely wrap ITK and VTK, which makes the total module base very comprehensive. MeVisLab has been evaluated as a very good platform for creating application prototypes using visual data-flow programming [25], is very well documented and supported by an active online community.

In MeVisLab, surface meshes are internally represented as Winged Edge Meshes (WEM) as proposed by Baumgart [26,27]. Each WEM in MeVisLab can consist of a number of WEM ''patches'', whereat each patch represents a closed set of ''faces'' that in total form the surface of a 3D model. These faces can be polygonal, but triangles are preferred and recommended. The standard distribution of MeVisLab contains about 4 dozens of pre-defined modules for creating, rendering, loading, saving and manipulating WEMs, including the ''WEMIsoSurface'' module that can be directly used to create a surface mesh out of a DICOM image (e.g. a segmentation mask).

The standard ''WEMSave'' module of MeVisLab provides the possibility to store WEM meshes in different formats, i.a. the popular STL format (STereoLithography format [28], also known as Standard Tessellation Language) into a file, but meta data besides the pure surface geometry is exported only for the proprietary binary Winged Edge Mesh format.

To overcome this lack, a new export module named ''WEMSaveAsU3D'' was created. Since U3D files can contain very detailed information about objects and the whole scene, a functional extension of the existing WEMSave module that predominantly only stores geometry data was not reasonable. As all modules for MeVisLab, the WEMSaveAsU3D was written in C++. Microsoft Visual Studio 2008 was used for editing and compiling the source code, as well as for debugging. The module class inherits from the ''WEMInspector'' base class since it serves as final module in a WEM processing chain.

To simplify the adding of new features, a set of tool methods was implemented and the complete set of constant definitions (e.g. material attributes and block type codes) of the ECMA-363 Standard were made available in a dedicated C++ Header file (WEMSaveAsU3D_Definitions.h, Figure 1).

The source code was verbosely annotated to facilitate programmers to understand and expand the implementation. Almost every line of code that is directly related to the U3D standard has a comment pointing to the respective chapter of the ECMA-363 document (Figure 1).

An additional module named ''ComposeWEMDescriptionForU3D'' was created to facilitate the user-friendly generation of meta data necessary for coloring and naming U3D objects. This module was implemented as a MeVisLab Macro Module using Python as programming language by reason that it is not a time-critical module and way easier to modify and extend this way.

Results

The WEMSaveAsU3D module and an auxiliary ComposeWEMDescriptionForU3D module

The new WEMSaveAsU3D module that has recently been integrated into the standard distribution of MeVisLab saves WEMs that consist of triangle faces into U3D files as defined in Standard ECMA-363. If a WEM contains more than one patch, each patch is converted to a U3D object. Therefore, each patch should have a unique name, specified by its ''Label'' property. If the names of the patches in a WEM are not unique (or not specified at all), the module creates new (unique) names for the U3D file. Within the U3D file, each U3D object carries the name (label) of the WEM patch it was created from.

More U3D object properties can be specified using the ''Description'' property of a WEM patch: the color (including transparency) of a single object and of an object group, the reflective color of an object, the name of an object group and the name of the overall model. These additional U3D properties need to be composed to a single string and thereafter written to the ''Description'' property of a WEM patch to be parsed by the module. The helper module ComposeWEMDescriptionForU3D facilitates the generation of valid string encoded U3D properties.

The current version of the WEMSaveAsU3D module does not implement all U3D features of the ECMA-363 standard. It is limited to triangle meshes, coloring, lighting and grouping of objects into a tree hierarchy. The missing features are discussed below.

Usage of the Modules

A detailed description of both modules and their usage is available with the MeVisLab documentation as well as online (http://www.mevislab.de/docs/current/MeVisLab/Standard/Documentation/Publish/ModuleReference/WEMSaveAsU3D.html).

Figure 2 shows the basic usage of the two modules; the MeVisLab network in the upper part (A) is the standard example network for the WEMSaveAsU3D module and implements the simplest processing chain: loading of a mesh, modifying the U3D properties and saving the U3D file. For MeVisLab novices, we strongly recommend reading the ''Getting Started'' tutorial mentioned above to understand how to create and work with a MeVisLab network. For a quick assessment of our modules, follow the instructions in Figure 3 to reproduce and use this example network.

```
// File structure blocks (section 9.4 of the Standard)

const MLuint32 U3D_BLOCKTYPE_FILEHEADER                    = 0x00443355;  // 9.4.1
const MLuint32 U3D_BLOCKTYPE_FILEREFERENCE                 = 0xFFFFFF12;  // 9.4.2
const MLuint32 U3D_BLOCKTYPE_MODIFIERCHAIN                 = 0xFFFFFF14;  // 9.4.3
const MLuint32 U3D_BLOCKTYPE_PRIORITYUPDATE                = 0xFFFFFF15;  // 9.4.4
const MLuint32 U3D_BLOCKTYPE_NEWOBJECTTYPE                 = 0xFFFFFF16;  // 9.4.5
```

Figure 1. Code snippet of pre-defined constants. This code snippet from WEMSaveAsU3D_Definitions.h shows comments pointing to the chapters of the ECMA-363 standard where the respective block type constants are defined.
doi:10.1371/journal.pone.0079004.g001

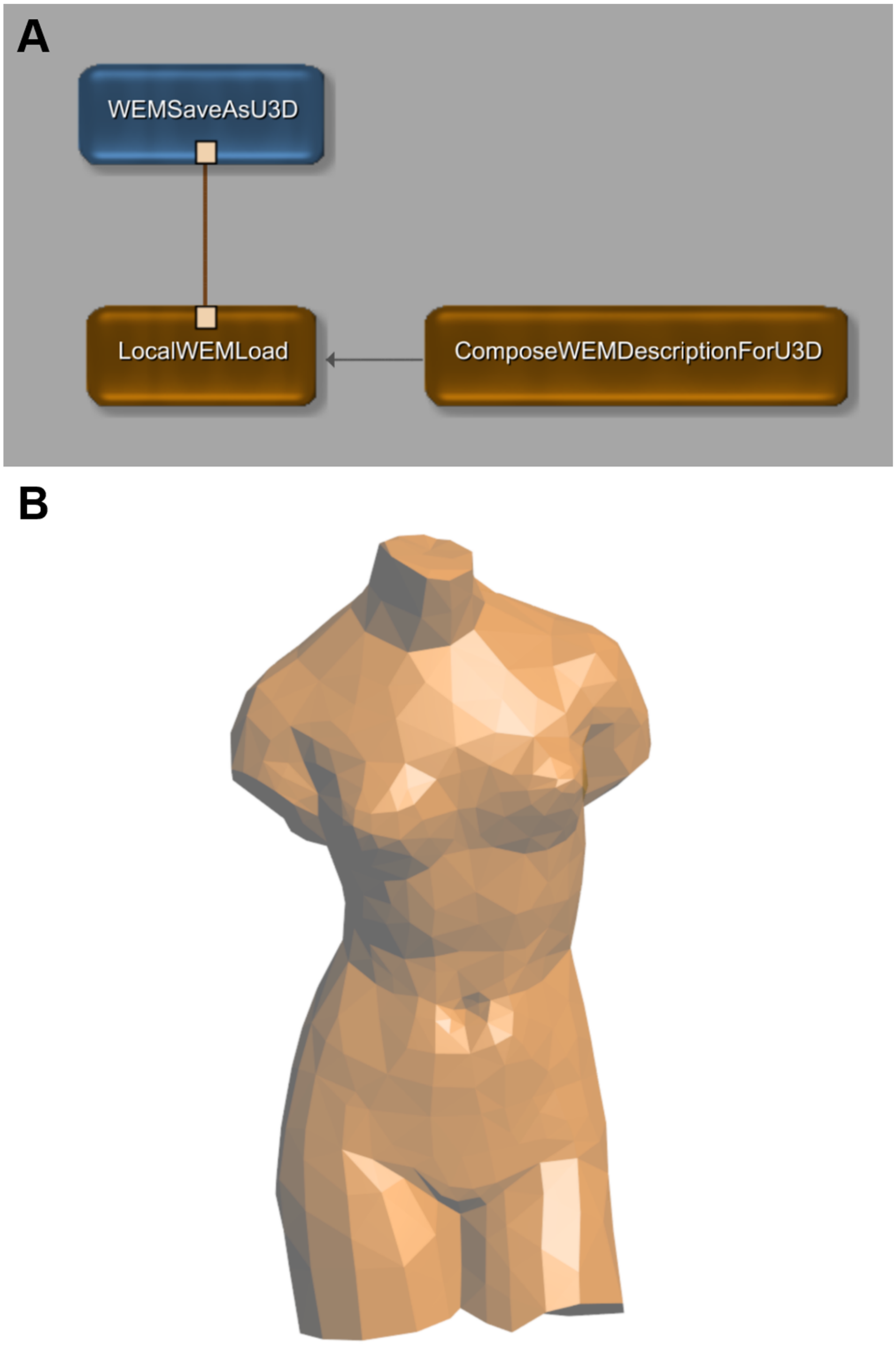

Click here to enable interactive mode.

Figure 2. Example Network for the new MeVisLab modules. (A) This example network illustrates the basic usage of the WEMSaveAsU3D module and the ComposeWEMDescriptionForU3D module. The network is available with the standard distribution of MeVisLab (right-click on the instance of a WEMSaveAsU3D module and select ''Show Example Network''). The LocalWEMLoad module loads a 3D model defined in Object File Format (''venus. off'', part of the MeVisLab demo data) and the WEMSaveAsU3D modules writes it into a U3D file. The ComposeWEMDesription-ForU3D module sets the color of the model as well as object and group names. The result is displayed on the bottom (B).
doi:10.1371/journal.pone.0079004.g002

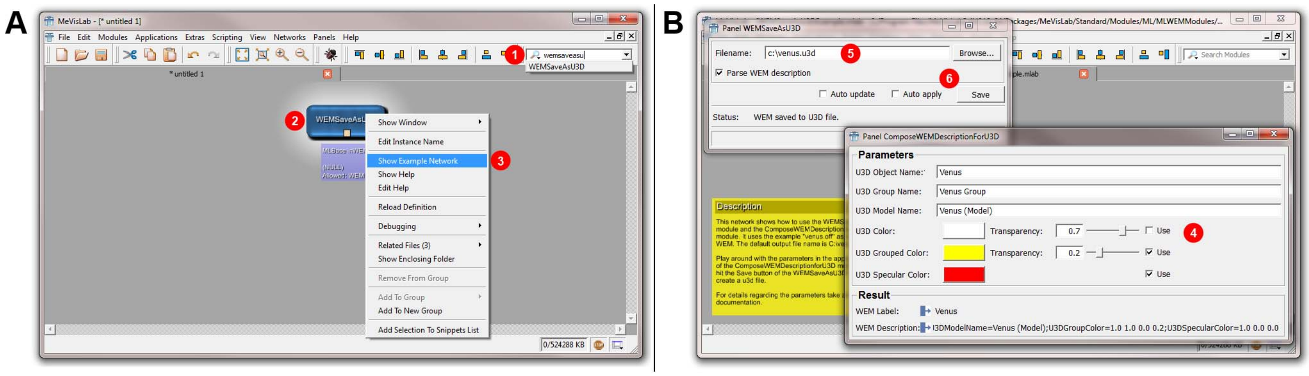

Figure 3. Quick reference to creating and using the modules. These screenshots illustrate how to create and use the modules for U3D export. 1. (A) Create a new network (Menu "File" →"New"). 2. (A) Create an instance of the WemSaveAsU3D module (type the name into the "Search Modules" field (1) and hit Enter). The module icon (2) should appear in the workspace. 3. (A) Open the example network of the module (right-click the module icon (2) and select "Show Example Network" (3) from the context menu). 4. (B) A new network tab and two module panels should open automatically. (If not, open the panels manually by double-clicking the module icons of WemSaveAsU3D and ComposeWEMDescriptionForU3D.) 5. (B) Modify the U3D model properties using the ComposeWEMDescriptionForU3D panel (4). 6. (B) To save the U3D file, go to the WemSaveAsU3D panel, specify a file name (5) and click "Save" (6). Other surface models (e.g. in the popular STL format) can be loaded by means of the LocalWEMLoad module (double-click the respective module icon and select the "Browse" button from the module panel).
doi:10.1371/journal.pone.0079004.g003

Figure 4 shows a more complex processing chain. The corresponding network is provided as File S1. Figure 5 gives an impression of a human femur, that has been segmented with MeVisLab and exported to U3D using various names and colors.

Discussion

A Simple and Straight Forward Way to Create 3D Model Data for Embedding in PDF

The WEMSaveAsU3D module for MeVisLab offers a simple way to create U3D files from surface meshes of biomedical data. It works "out-of-the-box" and comes with the standard MeVisLab distribution. The creation of the surface mesh itself can be completely handled within MeVisLab; the result can be exported directly into a U3D file. MeVisLab is available for free and for all three major platforms (Windows, Mac OS and Linux). By using MeVisLab for the generation of U3D model data, the direct export function of 3D content into PDF as demanded by conclusion #3 in [8] is almost fulfilled. The WEMSaveAsU3D module does not create a PDF file, but a U3D file with all necessary meta data for a direct import using PDF authoring software. The replacement of this authoring step seems not reasonable since 3D content will probably never be the only content of a PDF. By combining free PDF authoring tools like LaTeX (http://www.latex-project.org/) or the iText library (http://itextpdf.com/) with U3D models coming from MeVisLab, the complete PDF authoring process can be performed without any commercial software and on any of the major platforms.

The generation of the model surface still has to be done, but that is also a step MeVisLab can be used for. More than 2600 image processing modules (including ITK and VTK) provide a vast potential of finding a solution for many biomedical image processing and analysis challenges.

To give an example: all four software applications used for segmentation & surface mesh generation (Mimics by Materialise), scene assembling (Maya by Alias), object categorization (Deep Exploration Standard by Right Hemisphere) and coloring (3D Reviewer by Adobe) for the Visible Korean Project [12] could probably have been replaced by a single MeVisLab image processing network, thus avoiding the use of intermediate file formats (MCS, STL, VRML) and workflow discontinuity (see Figure 5 for a segmentation result example).

And even if the problem itself has already been solved by another software, MeVisLab and the WEMSaveAsU3D module can still be used to convert existing model data into U3D if the model surface is available in a popular alternative format (STereoLithography/Standard Tessellation Language, Object File Format, Wavefront or Polygon File Format). MeVisLab also offers the possibility to convert Open Inventor Scenes into WEMs which then can be exported into U3D as shown in Figure 4.

Further Development

There are four U3D features of minor importance for biomedical imaging still missing as regards WEM export from MeVisLab: labeling, textures, alternative geometry definition (point clouds & line set) and pre-defined animations.

The possibility to embed 3D labels ("2D Glyphs" in U3D terminology, demonstrated in fig. 2 and fig. 7 of [29]) makes it easy to clearly identify objects within the space of an interactive 3D scene, independently from the view selected by the user. Especially for PDFs with educational purpose as discussed in [12], e.g. for teaching anatomy to medical students, an undoubtful labeling of structures with complex spatial relationships can be very serviceable.

Application of textures to 3D models (e.g. a human face as demonstrated in Additional File #1 of [10]), is of limited utility, except for a simulated volume rendering as shown in fig. 5 of [29]. The main disadvantages of this simulated volume rendering are fixed windowing and file size. The rendering can be embedded with only one pre-defined window setting that must match the preference and intent of the viewer. In Addition to that, a complete set of textured slices for each of the three Cartesian axes must be embedded, which inflates the file size. On the other hand, simulated volume rendering within PDF documents offers a new way of publishing biomedical 3D images.

U3D models can be defined as point clouds (fig. 1 of [29]) or line sets. The latter could be used for visualization of vessel centerlines, nervous fiber tracking or 3D ECG diagrams. Fig. 2(B) of [30] is a good example of 3D ECG data visualization

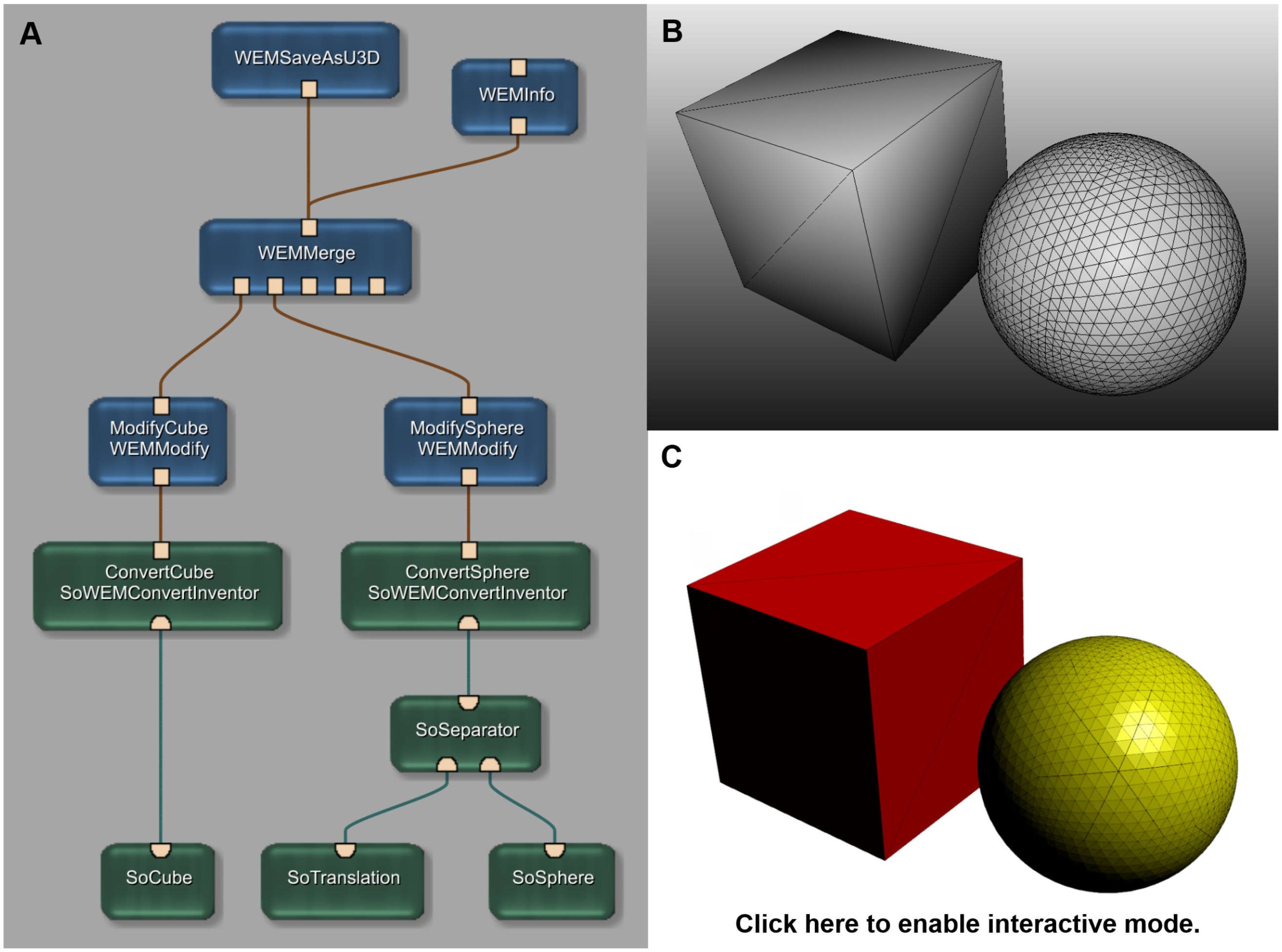

Figure 4. Example of a more complex application network. (A) This example network simulates a complex image processing chain (read from bottom to top). The network generates an Open Inventor Scene with a cube and a sphere as "segmentation results" (B). The two objects are then converted into WEM patches (SoWEMConvertInventor modules) and the properties (names and colors) are set (WEMModify modules). Finally the two WEM patches are merged into one WEM and afterwards written into a U3D file. The result is displayed on the bottom right (C). A file containing this network is provided as File S1.
doi:10.1371/journal.pone.0079004.g004

constricted by representation in a 2D figure that should ideally be presented as a 3D model to reveal the full information content.

The last missing feature of U3D is pre-defined animation ("motion" in U3D terms) which is limited to rotation and translation - a model deformation is not possible. This makes it impossible to display e.g. the dynamics of a beating heart whereas the animation of moving joints and their adjacent bones is conceivable, e.g. for educational purposes.

Although the currently available version of the WEMSaveAsU3D module cannot utilize any of the previously discussed U3D features, their implementation is planned for future releases. Since the source code of the module is verbosely commented and available with the MeVisLab distribution since version 2.4, the implementation can also be done by any user with sufficient C++ programming skills. All necessary tool methods and constants for writing the respective U3D Modifier Blocks and Resource Blocks (chapter 9.7 and 9.8 of [21]) are already implemented and used by the current version of the module. The source code can be found after the complete installation of MeVisLab in [Install Path]/Packages/MeVisLab/Standard/Sources/ML/MLWEMModules/WEMSaveAsU3D.

The DICOM Supplement 132 [31] defines a Surface Segmentation Storage SOP Class based on triangle meshes. Although MeVisLab currently does not comprise an import module for DICOM Supplement 132 files, it is desirable to add one as heir to the "WEMGenerator" base class. Once implemented, such a module would close the gap between generic DICOM segmentation results stored as surface meshes and their conversion into U3D files for embedding into PDF.

File Size Considerations

The last and probably most important issue regarding U3D data and the respecting PDF files incorporating them is the overall file size. 3D model data can be very large: one of the results of the Visible Korean Project [12], a highly detailed 3D PDF of a male head has a size of almost 100 MiBytes even though the raw data has been reduced reasonably. To achieve the smallest possible file size while preserving the most of the comprising information, an intelligent reduction of the number of surface triangles is inevitable. The "WEMReducePolygons" module of MeVisLab allows for reducing the number triangles by collapsing edges using a Quadric Error Metric [32]. Each of these collapse operations

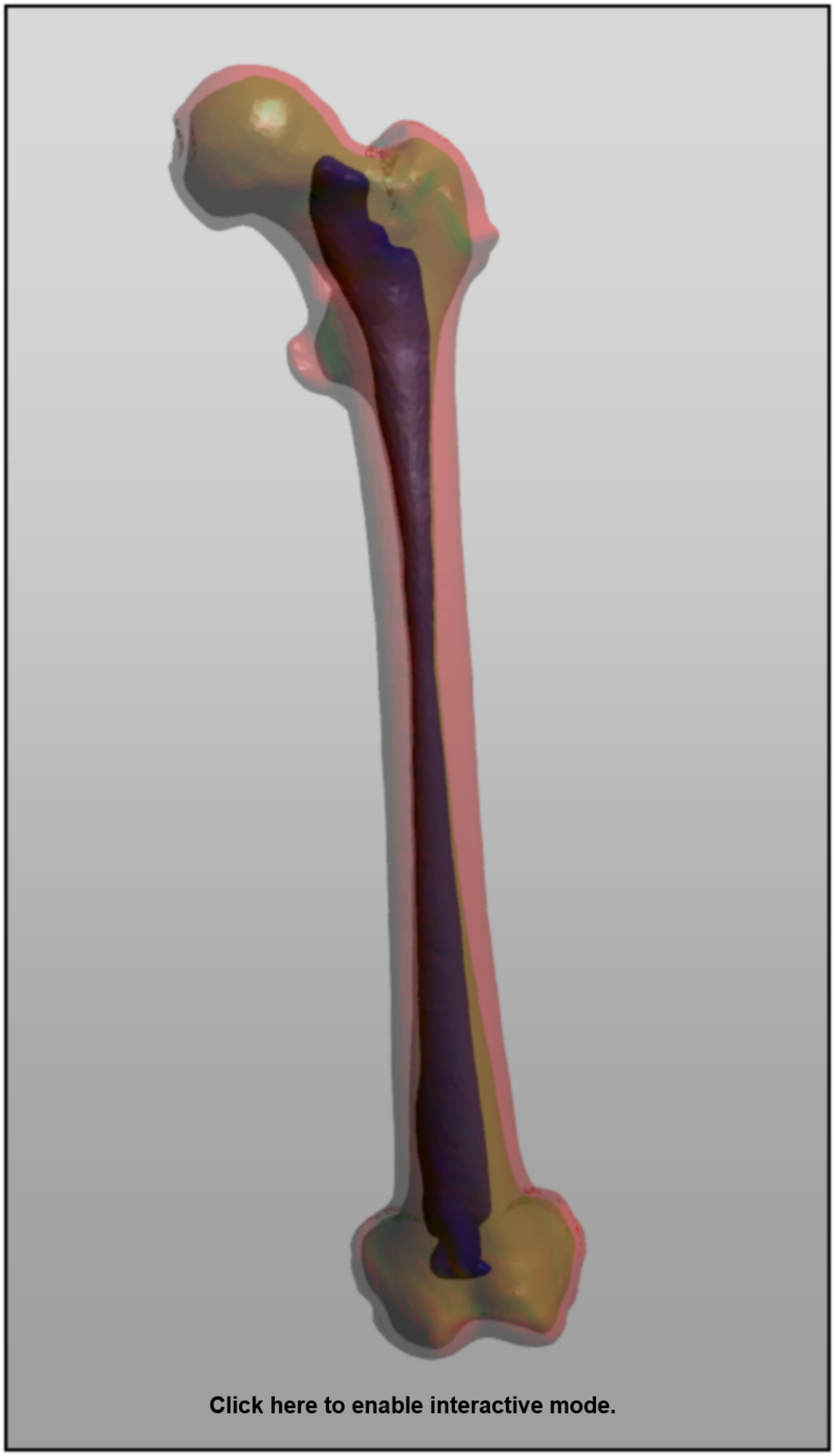

Figure 5. Model of a segmented femur. Model of a left human femur segmented with MeVisLab. The model shows the outer surface (red), the surface between compact bone and spongy bone (green) and the surface of the bone marrow (blue).
doi:10.1371/journal.pone.0079004.g005

introduces an error in the resulting mesh. Edges that cause as little error as possible are collapsed first thus preserving as much of the original shape as possible. Using this method, triangles defining plane surfaces have highest priority to be replaced by a more coarse mesh. A good example for a reasonable application of this triangle reduction strategy is the orthodontic model embedded in fig. 1 of [8]: the top face of the model is composed of hundreds of triangles that could be reduced to a number of only 7 without

losing any information. The creator of the final model has to make a tradeoff between model details and file size, but in most cases the number of triangles can be reduced by a large percentage without losing substantial information while greatly reducing file size. From our experience, a reduction rate of 95% (based on a voxel-precise mesh) is acceptable for most illustrational purposes and was applied for Figure 5. Regarding modern broadband internet connections and network speed, file sizes of around 10 MiBytes should not be a problem.

Conclusion

Modern science produces data with three-dimensional nature in many disciplines. PDF technology offers the possibility to publish this data in all its dimensions and should therefore be used accordingly. With MeVisLab and only one additional PDF authoring tool, the complete process of generating 3D PDF documents for biomedical publications can be handled in a consolidated working environment, free of license costs and with all major operating systems. The new WEMSaveAsU3D module does not feature all capabilities of the U3D standard, but covers most of the current use cases for 3D visualization in the biomedical domain. Due to the availability of the well documented source code, additional features can be added with low effort if needed.

Supporting Information

File S1 MeVisLab network file of the image processing chain shown in Figure 4 (A).
(MLAB)

File S2 Supplementary version of this article with embedded 3-d figures.
(PDF)

Author Contributions

Conceived and designed the experiments: AN. Wrote the paper: AN TG. Implemented the software: AN.

References

1. Adobe Systems Incorporated (1985–2004) PDF Reference, fifth edition, Adobe Portable Document Format, Version 1.6. Available: http://wwwimages.adobe.com/www.adobe.com/content/dam/Adobe/en/devnet/pdf/pdfs/pdf_reference_archives/PDFReference16.pdf. Accessed 10 June 2013.
2. Ruthensteiner B, Heß M (2008) Embedding 3D Models of Biological Specimens in PDF Publications. Microsc Res Tech 71: 778–786.
3. Iwasa JH (2010) Animating the model figure. Trends Cell Biol 20: 699–704.
4. Kumar P, Ziegler A, Ziegler J, Uchanska-Ziegler B, Ziegler A (2008) Grasping molecular structures through publication-integrated 3D models. Trends Biochem Sci 33: 408–412.
5. Selvam L, Vasilyev V, Wang F (2009) Methylation of zebularine a quantum mechanical study incorporating interactive 3D pdf graphs. J Phys Chem B 113: 11496–11504.
6. Kumar P, Ziegler A, Grahn A, Hee CS, Ziegler A (2010) Leaving the structural ivory tower, assisted by interactive 3D PDF. Trends Biochem Sci 35: 419–422.
7. Vasilyev V (2010) Towards interactive 3D graphics in chemistry publications. Theor Chem Acc 125: 173–176.
8. Danz JC, Katsaros C (2011) Three-dimensional portable document format: a simple way to present 3-dimensional data in an electronic publication. Am J Orthod Dentofacial Orthop 140: 274–276.
9. de Boer BA, Soufan AT, Hagoort J, Mohun TJ, van den Hoff MJB, et al. (2011) The interactive presentation of 3D information obtained from reconstructed datasets and 3D placement of single histological sections with the 3D portable document format. Development 138: 159–167.
10. Ziegler A, Mietchen D, Faber C, von Hausen W, Schöbel C, et al. (2011) Effectively incorporating selected multimedia content into medical publications. BMC Med 9: 17.
11. Phelps A, Naeger DM, Marcovici P (2012) Embedding 3D radiology models in portable document format. Am J Roentgenol 199: 1342–1344.
12. Shin DS, Chung MS, Park JS, Park HS, Lee S, et al. (2012) Portable document format file showing the surface models of cadaver whole body. J Korean Med Sci 27: 849–856.
13. Herfarth C, Lamadé W, Fischer L, Chiu P, Cardenas C, et al. (2002) The Effect of Virtual Reality and Training on Liver Operation Planning. Swiss Surg 8: 67–73.
14. Maunsell J (2010) Announcement Regarding Supplemental Material. J Neurosci 30: 10599–10600.
15. Thoma GR, Ford G, Antani S, Demner-Fushman D, Chung M, et al. (2010) Interactive Publication: The document as a research tool. J Web Semant 8: 145–150.
16. International Organization for Standardization (ISO) (2008) ISO 32000–1: 2008 Document management – Portable document format – Part 1: PDF 1.7. Available: http://www.iso.org/iso/iso_catalogue/catalogue_tc/catalogue_detail.htm?csnumber = 51502. Accessed 10 June 2013.
17. Castiglione A, De Santis A, Soriente C (2010) Security and privacy issues in the Portable Document Format. J Syst Software 83: 1813–1822.
18. Association for Information and Image Management (2008) BEST PRACTICES - IMPLEMENTATION GUIDE FOR THE PORTABLE DOCUMENT FORMAT HEALTHCARE. Available: http://www.aiim.org/documents/standards/PDF-h_Implementation_Guide_2008.pdf. Accessed 10 June 2013.
19. Adobe Systems Incorporated (2007) Press release: Adobe to Release PDF for Industry Standardization. Available: http://www.adobe.com/de/aboutadobe/pressroom/pr/jan2007/OpenPDF.pdf. Accessed 10 June 2013.
20. DICOM Standards Committee, Working Group 6 (2005) Digital Imaging and Communications in Medicine (DICOM) Supplement 104: DICOM Encapsulation of PDF Documents. Available: ftp://medical.nema.org/medical/dicom/final/sup104_ft.pdf. Accessed 10 June 2013.
21. ECMA International (2007) Standard ECMA-363, Universal 3D File Format, 4th edition (June 2007) Available: http://www.ecma-international.org/publications/files/ECMA-ST/ECMA-363%204th%20Edition.pdf.Accessed 10 June 2013.
22. Koenig M, Spindler W, Rexilius J, Jomier J, Link F, et al. (2006) Embedding VTK and ITK into a visual programming and rapid prototyping platform. Proc. Medical Imaging 2006: Visualization, Image-Guided Procedures, and Display 6141.
23. Heckel F, Schwier M, Peitgen HO (2009) Object oriented application development with MeVisLab and Python. Lecture Notes in Informatics (Informatik 2009: Im Focus das Leben) 154: 1338–1351.
24. Ritter F, Boskamp T, Homeyer A, Laue H, Schwier M, et al. (2011) Medical image analysis. IEEE Pulse 2: 60–70.
25. Bitter I, Van Uitert R, Wolf I, Ibanez L, Kuhnigk JM (2007) Comparison of four freely available frameworks for image processing and visualization that use ITK. IEEE T Vis Comput Gr 13: 483–493.
26. Baumgart BG (1972) Winged edge polyhedron representation. Available: http://www.dtic.mil/cgi-bin/GetTRDoc?Location = U2&doc = GetTRDoc.pdf&AD = AD0755141. Accessed 10 June 2013.
27. Baumgart BG (1975) A polyhedron representation for computer vision. In: Proc. May 19–22, 1975, national computer conference and exposition, ACM: 589–596.
28. Burns M (1993) The StL Format. In: Burns M. Automated fabrication: improving productivity in manufacturing. Prentice-Hall, Inc.
29. Barnes DG, Fluke CJ (2008) Incorporating interactive 3-dimensional graphics in astronomy research papers. New Astron 13: 599–605.
30. Tereshchenko LG, Han L, Cheng A, Marine JE, Spragg DD, et al. (2010) Beat-to-beat three-dimensional ECG variability predicts ventricular arrhythmia in ICD recipients. Heart Rhythm 11: 1606–1613.
31. DICOM Standards Committee, Working Group 6 (2008) Digital Imaging and Communications in Medicine (DICOM) Supplement 132: Surface Segmentation Storage SOP Class. Available: ftp://medical.nema.org/medical/dicom/final/sup132_ft.pdf. Accessed 10 June 2013.
32. Garland M, Heckbert PS (1997) Surface simplification using quadric error metrics. Proc. 24th Annual Conference on Computer Graphics and Interactive Techniques: 209–216.
